# Speaker-turn aware diarization for speech-based cognitive assessments

**DOI:** 10.3389/fnins.2023.1351848

**Published:** 2024-01-16

**Authors:** Sean Shensheng Xu, Xiaoquan Ke, Man-Wai Mak, Ka Ho Wong, Helen Meng, Timothy C. Y. Kwok, Jason Gu, Jian Zhang, Wei Tao, Chunqi Chang

**Affiliations:** ^1^School of Biomedical Engineering, Shenzhen University Medical School, Shenzhen University, Shenzhen, China; ^2^Department of Electronic and Information Engineering, The Hong Kong Polytechnic University, Kowloon, Hong Kong SAR, China; ^3^Department of Systems Engineering and Engineering Management, The Chinese University of Hong Kong, Shatin, Hong Kong SAR, China; ^4^Department of Medicine and Therapeutics, The Chinese University of Hong Kong, Shatin, Hong Kong SAR, China; ^5^Jockey Club Centre for Osteoporosis Care and Control, The Chinese University of Hong Kong, Shatin, Hong Kong SAR, China; ^6^Department of Electrical & Computer Engineering, Dalhousie University, Halifax, NS, Canada; ^7^School of Pharmacy, Shenzhen University Medical School, Shenzhen University, Shenzhen, China; ^8^Department of Neurosurgery, South China Hospital of Shenzhen University, Shenzhen, China

**Keywords:** speaker diarization, speaker embedding, comprehensive scoring, speaker-turn timestamps, MOCA, dementia detection

## Abstract

**Introduction:**

Speaker diarization is an essential preprocessing step for diagnosing cognitive impairments from speech-based Montreal cognitive assessments (MoCA).

**Methods:**

This paper proposes three enhancements to the conventional speaker diarization methods for such assessments. The enhancements tackle the challenges of diarizing MoCA recordings on two fronts. First, multi-scale channel interdependence speaker embedding is used as the front-end speaker representation for overcoming the acoustic mismatch caused by far-field microphones. Specifically, a squeeze-and-excitation (SE) unit and channel-dependent attention are added to Res2Net blocks for multi-scale feature aggregation. Second, a sequence comparison approach with a holistic view of the whole conversation is applied to measure the similarity of short speech segments in the conversation, which results in a speaker-turn aware scoring matrix for the subsequent clustering step. Third, to further enhance the diarization performance, we propose incorporating a pairwise similarity measure so that the speaker-turn aware scoring matrix contains both local and global information across the segments.

**Results:**

Evaluations on an interactive MoCA dataset show that the proposed enhancements lead to a diarization system that outperforms the conventional x-vector/PLDA systems under language-, age-, and microphone-mismatch scenarios.

**Discussion:**

The results also show that the proposed enhancements can help hypothesize the speaker-turn timestamps, making the diarization method amendable to datasets without timestamp information.

## 1 Introduction

Mild cognitive impairment (MCI) is a memory and cognitive impairment stage in which patients may notice that their memory or mental function has declined, although the impairment does not significantly affect their daily activities. Studies have shown that patients with MCI are at high risk of having dementia, which can quickly develop into Alzheimer's disease (AD; Kantarci et al., 2009). Alzheimer's Disease International (ADI) reported in 2015 that there were up to 46 million people with dementia in the world, and this number is predicted to increase to more than 130 million by 2050 (Prince, 2017). Although no evidence-based medications can be recommended for the MCI patients (Cooper et al., 2013), the screening and early identification of the patients with cognitive impairment is one of the most critical determinants for preventing and treating dementia and AD.

Cognitive tests are tools for assessing human cognitive capabilities, with notable examples including Mini-Mental State Exam (MMSE; Pangman et al., 2000), Mini-Cognitive scale (Mini-Cog; Borson et al., 2000), and Montreal Cognitive Assessment (MoCA; Nasreddine et al., 2005). Each of these tests has specific applications and has been validated for various cognitive assessments. The MMSE has a short assessment time (5–10 min). During an MMSE session, a participant is asked to say the date, count backward, and identify objects in a room. Although MMSE is simple to use, it lacks the sensitivity to detect MCI (Chapman et al., 2016). The Mini-Cog is a fast (3 min) cognitive assessment and is more acceptable by participants. It involves memorizing and recounting a short list of objects and making a drawing. It is mainly used for detecting dementia, but there is insufficient evidence to recommend or against using Mini-Cog as a cognitive screen tool in community settings (Fage et al., 2015). The MoCA takes longer and covers more domains than the MMSE and Mini-Cog, resulting in higher sensitivity and specificity (Ciesielska et al., 2016). This leads to its frequent application in the detection of MCI and AD among the elderly.

Studies have found that the irregularities appeared in patients' speech and connected language could be due to MCI and AD (Konig et al., 2018; Mueller et al., 2018), which calls for using spoken language technologies to detect MCI and AD from patients' MoCA recordings. Because a MoCA session involves the spoken dialogs between an assessor and a patient, it is essential to perform speaker diarization to extract the utterances spoken by the patient as a first step toward the efficient analysis of the patient's speech.

**Figure 1:**
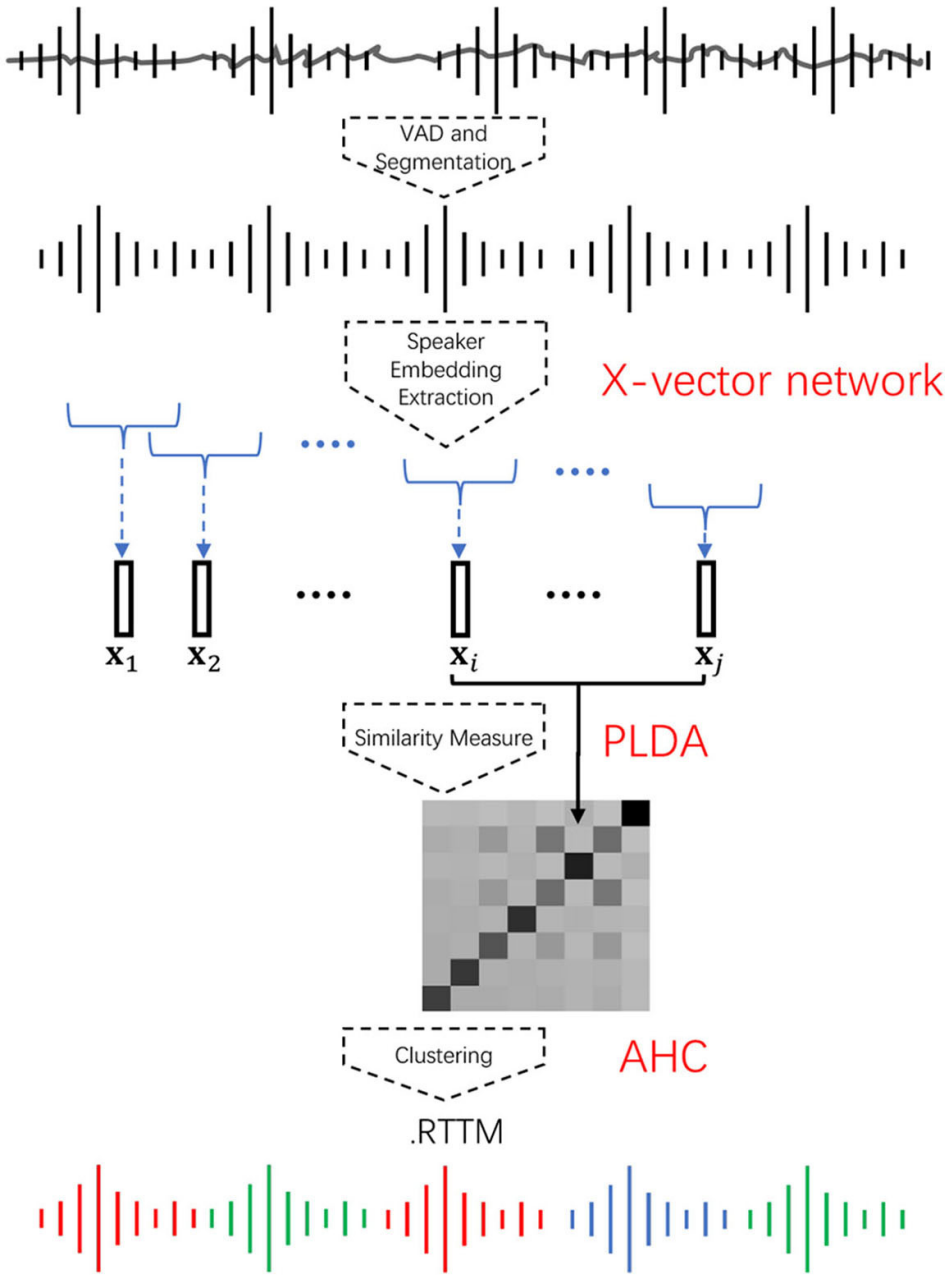
A standard x-vector–PLDA–AHC speaker diarization framework. The speech parts of the audio are extracted as short segments. The segments are embedded into a space representing the speakers' characteristics. The similarity between every pair of segments is then computed to form a similarity matrix, which is then used for agglomerative hierarchical clustering to determine the timestamps of individual speaker turns.

Speaker diarization is the process of partitioning an input audio into homogeneous segments according to the speaker identities. It answers the question of who spoke when. Figure 1 outlines a standard diarization framework. In general, the diarization process consists of the following steps. First, a voice activity detector (VAD) is applied to remove non-speech parts from the input audio. Next, speech regions are uniformly partitioned into short overlapping segments. After that, the segments are mapped to a fixed-dimensional feature space by a speaker embedding network such as the x-vector network (Garcia-Romero et al., 2017; Snyder et al., 2018). Then, a similarity matrix is produced by computing the PLDA score (Prince and Elder, 2007; Mak and Chien, 2020) of every segment pair. Finally, agglomerative hierarchical clustering (AHC) is applied to the similarity matrix to obtain a rich transcription time mark (RTTM) (Fiscus et al., 2005) format file. The RTTM contains the speaker identity of each turn and its corresponding timestamps, i.e., information about when an identified speaker began speaking and how long the speaker spoke. In other words, given an audio and the corresponding RTTM, the audio segments of different speakers can be extracted. A basic problem is how to embed the speech segments such that different speakers can be embedded into different regions of the embedding space, regardless of the speech content. In this work, we propose enhancing the embedding extraction process, the similarity measures, and the clustering process in the conventional x-vector–PLDA–AHC pipeline.

**Figure 2:**
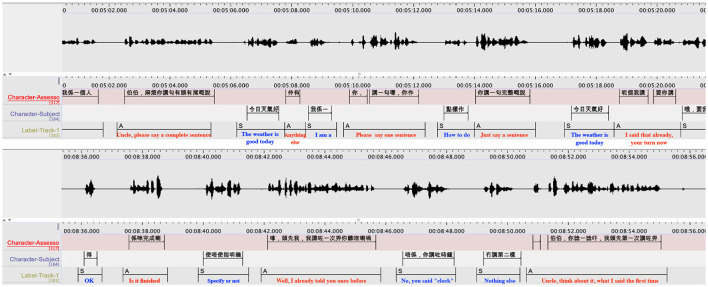
Recordings of a Cantonese MoCA session with the diarization results of a dialogue between an assessor and a participant. In the figures, the **(top)** panel is speech waveform. The ground truth transcripts are shown in the **(middle)** panel. The outputs of speaker diarization are shown in the **(bottom)** panel, where “A” refers to the assessor and “S” refers to the subject.

The MoCA recordings present special challenges to speaker diarization. Conventionally, researchers of speaker embeddings focused on long utterances (over 5 s) due to the large variance in the x-vectors representation under short-utterance scenarios. However, the MoCA tests consist mainly of short utterances in interactive dialogs. Figure 2 gives an example of Cantonese-based MoCA recordings with diarization results. It is difficult to extract sufficient information to discriminate speakers under short-utterance scenarios. This problem is exacerbated by the fact that the interactive dialogs have backchannel cues and frequent changes in speaker turns, which lead to a high probability of missing the speaker change points. An essential requirement of MoCA tests is that the recording devices should not disturb or affect the patient during a recording session. Ideally, the patient should not aware the existence of the devices. Therefore, in practice, MoCA sessions use far-field microphones for recording. But this will cause microphone mismatch because diarization systems are typically trained on speech recorded by close-talking microphones. The mismatch calls for a more robust speaker embedding method that is less sensitive to the microphone types.

Agglomerative hierarchical clustering (Luxburg, 2007) is a notable clustering approach to speaker diarization. Bayesian information criterion is usually used to estimate which couple of clusters should be merged at each agglomerative iteration. This leads to a high computational cost when the number of data points increases. Also, the performance of AHC heavily depends on the choice of the distance metric (Han et al., 2008). In contrast, spectral clustering (SC; Luxburg, 2007) does not require a statistical metric to determine whether two clusters should be merged. Previous researches have applied SC to infer speaker clusters and achieved good performance (Iso, 2010; Ning et al., 2010), especially in speaker diarization tasks (Ning et al., 2006).

This work aims to enhance our age-invariant diarization system in Xu et al. (2021a) and our scoring method for diarization in Xu et al. (2021b) for speech-based cognitive assessments. A speaker embedding extractor, CE-Res2Net (Zhao and Mak, 2021), is used to produce multi-scale channel interdependence speaker embeddings as front-end representations. Instead of PLDA, an LSTM-based scoring model (Lin et al., 2019) trained on the sequential information across short speech segments is applied for similarity measure. Moreover, a comprehensive similarity measure is proposed to enhance the diarization performance further. The measure, a weighted sum of an LSTM-based similarity score and a cosine-distance score, enables the speaker-embedding network to capture the local sequential structure of the speech segments and their global similarity. The resulting diarization system was applied to a MoCA dataset comprising 469 older adults, including healthy individuals and patients with mild to major neurocognitive disorders (NCDs). It was found that the enhanced embedding can overcome the acoustic mismatch due to the far-field microphones, that the LSTM-based model can leverage the ground-truth speaker-turn information in the training data or the hypothesized timestamps in the MoCA data, and that the proposed comprehensive scoring can capture both local and global information across segments.

From a practical perspective, the proposed diarization method can serve as a front-end module for spoken-language-based AD screening tools. Such tools can help clinicians quickly identify early AD patients for further diagnosis. From a machine learning perspective, the contributions include applying multi-scale channel interdependence speaker embedding to model the local dependence within short speech segments and to overcome the acoustic mismatch in clinic environments. A sequence comparison approach is proposed to capture the long-term dependence in these segments for cognitive assessments. Moreover, a comprehensive similarity measure is proposed to consider both local and global information across the segments.

Conventional diarization systems are trained to identify multiple speakers in a conversation, known as speaker-aware. They do not leverage the sequence of speaker turns and their time stamp in the training data. The proposed approach goes a step further by being speaker-turn aware. This awareness means that the model can learn from the speaker-turn information in the training data to determine who spoke when in an unseen conversation. This is achieved by leveraging the time stamp information in the training data, which enables our system to understand the temporal aspects of the short segments in a conversation. The utilization of the time stamp information is one of the key advantages of our approach over traditional clustering-based diarization approaches. The approach makes the scoring matrix more relevant to the diarization task because it encapsulates information about who is speaking at what time and for how long. This property makes the proposed approach effective for analyzing speech in cognitive assessments.

## 2 Materials and methods

### 2.1 Diarization system overview

#### 2.1.1 Speaker embedding networks

X-vector is a speaker embedding approach based on deep neural networks (DNN), which has demonstrated good performance in both speaker recognition (Snyder et al., 2018) and speaker diarization (Garcia-Romero et al., 2017). In Kaldi's x-vector networks (Povey et al., 2011), MFCCs are extracted and fed to time-delay layers (Snyder et al., 2017) for frame-level processing. Then, a statistics pooling layer aggregates over the frame-level representations at the last time-delay layer into a segment-level representation, followed by two fully connected layers and a softmax layer to output the posterior probabilities of speakers. The penultimate layer's outputs form the speaker embeddings called x-vectors.

After the invention of x-vector, researchers have focused on enriching the speaker's information in the embedding vectors. The enhancements include ResNet embedding (Zeinali et al., 2019) that adds residual connections, DenseNet embedding (Lin et al., 2020) that leverages inter-layer connections, and ECAPA-TDNN (Desplanques et al., 2020) that models channel inter-dependencies and exploits multi-scale features. Also, the mean and standard deviation computation in the statistics pooling layer has been enhanced by an attention mechanism (Zhu et al., 2018; Lin and Mak, 2022).

#### 2.1.2 Conventional similarity measures

Cosine similarity and probabilistic linear discriminant analysis (PLDA; Ioffe, 2006; Prince and Elder, 2007) are two popular approaches to measuring the similarities between speech segments in speaker diarization. Given an audio recording, the waveform is partitioned into many segments, typically 1.5 s with 0.75 s overlapping. Then, the speaker embedding vector of each segment is computed by presenting the acoustic vectors of the segment to the speaker embedding network. Each embedding vector is compared with all the others to form a similarity matrix, which is then past to a clustering algorithm (see Section 2.1.3) to group similar embedding vectors into speaker groups.

Cosine similarity measures the closeness between a pair of speaker embedding vectors (e.g., **x**_*i*_ and **x**_*j*_) by Equation (1), which is the cosine of the angle between the two vectors:


(1) $$\text{cosine}(x_{i},x_{j})=\frac{x_{i}\cdot x_{j}}{\left\|x_{i}\right\|\left\|x_{j}\right\|}.$$

A score close to 1.0 means that the two embeddings are similar. If the score is larger than a threshold θ, we consider the embeddings are from the same speaker.

PLDA is a factor analysis technique in which the variability of speaker embedding vectors can be separately modeled by a within-speaker covariance matrix and a between-speaker covariance matrix. The former characterizes non-speaker variability, whereas the latter captures speaker variability. By separating the speaker and non-speaker variabilities, a PLDA model can suppress the non-speaker variabilities when comparing the similarity between two speaker embeddings. Specifically, given a pair of speaker embeddings **x**_*i*_ and **x**_*j*_, the PLDA model with the model parameter ω computes a log-likelihood ratio (LLR) score based on the same-speaker hypothesis $H_{0}$ and different-speaker hypothesis $H_{1}$ (Mak and Chien, 2020), as in Equation (2):


(2) $$\begin{array}{l} S(x_{i},x_{j})=\log\frac{p(x_{i},x_{j}|{ℋ}_{0})}{p(x_{i},x_{j}|{ℋ}_{1})}\\ \;=\frac{\int p(x_{i},x_{j},z|\omega )\text{d}z}{\int p(x_{i},z_{i}|\omega )\text{d}z_{i}\int p(x_{j},z_{j}|\omega )\text{d}z_{j}}\\ \;=\frac{\int p(x_{i},x_{j}|z,\omega )p(z)\text{d}z}{\int p(x_{i}|z_{i},\omega )p(z_{i})\text{d}z_{i}\int p(x_{j}|z_{j},\omega )p(z_{j})\text{d}z_{j}}. \end{array}$$

Note that the speaker factor **z** is identical to both **x**_*i*_ and **x**_*j*_ in $H_{0}$, whereas they are different in $H_{1}$. For a Gaussian PLDA model, the LLR can be computed using Equation (3) (Garcia-Romero and Espy-Wilson, 2011):


(3) $$S(x_{i},x_{j})\;=\frac{1}{2}x_{i}^{T}Qx_{i}+x_{i}^{T}Px_{j}+\frac{1}{2}x_{j}^{T}Qx_{j}+\text{const},$$

where **P** and **Q** are trainable parameters and can be defined as in Equation (4),


(4) $$\begin{array}{l} \text{P}={\text{Λ}}^{-1}\text{Γ}(\text{Λ}-\text{Γ}{\text{Λ}}^{-1}\text{Γ})-1\\ \text{Q}={\text{Λ}}^{-1}-(\text{Λ}-\text{Γ}{\text{Λ}}^{-1}\text{Γ})-1, \end{array}$$

and **Λ** is calculated using Equation (5),


(5) $$\text{Λ}=\text{W}{\text{W}}^{\text{T}}+\text{ Σ},\;\text{Γ}=\text{W}{\text{W}}^{\text{T}}.$$

where **W** is the speaker loading matrix and **Σ** is the covariance matrix representing non-speaker variability. The scores derived from the PLDA model is considered as similarity scores, which can be used by clustering algorithms such as AHC.

Compared with the cosine similarity, a PLDA model has trainable parameters (e.g., **P** and **Q** in Equation 3) leveraging the speaker labels in the training data. Therefore, PLDA is more effective in discriminating speakers. However, its popularity waned with the advent of more advanced embedding networks (Xiang et al., 2019). In addition, cosine similarity and PLDA scoring assume that speaker embeddings are Gaussian distributed. Therefore, if the speaker embeddings are distributed irregularly, both back ends may exhibit performance degradation.

According to Equations (1) and (3), both the cosine similarity and PLDA do not consider the information from the neighborhoods of the two speech segments. In other words, they focus on the pairwise comparisons of the two segments rather than the temporal structure of the conversation.

#### 2.1.3 Agglomerative hierarchical clustering

The x-vector–PLDA–AHC framework has been used in various speaker diarization systems (Han et al., 2008; Diez et al., 2019; Xu et al., 2021a). AHC is an unsupervised clustering and merging method. To apply AHC, we performed PLDA scoring on all pairs of segments (x-vectors) for each recording. The PLDA scores were then used as input to the AHC algorithm for classifying speech segments by speaker identities. In this work, the baseline systems were conducted based on this framework.

AHC requires a similarity matrix comprising similarity scores among all speech segments in a conversation. In this work, AHC is based on PLDA scores.

### 2.2 Proposed diarization system

#### 2.2.1 Channel-interdependence enhanced Res2Net

**Figure 3:**
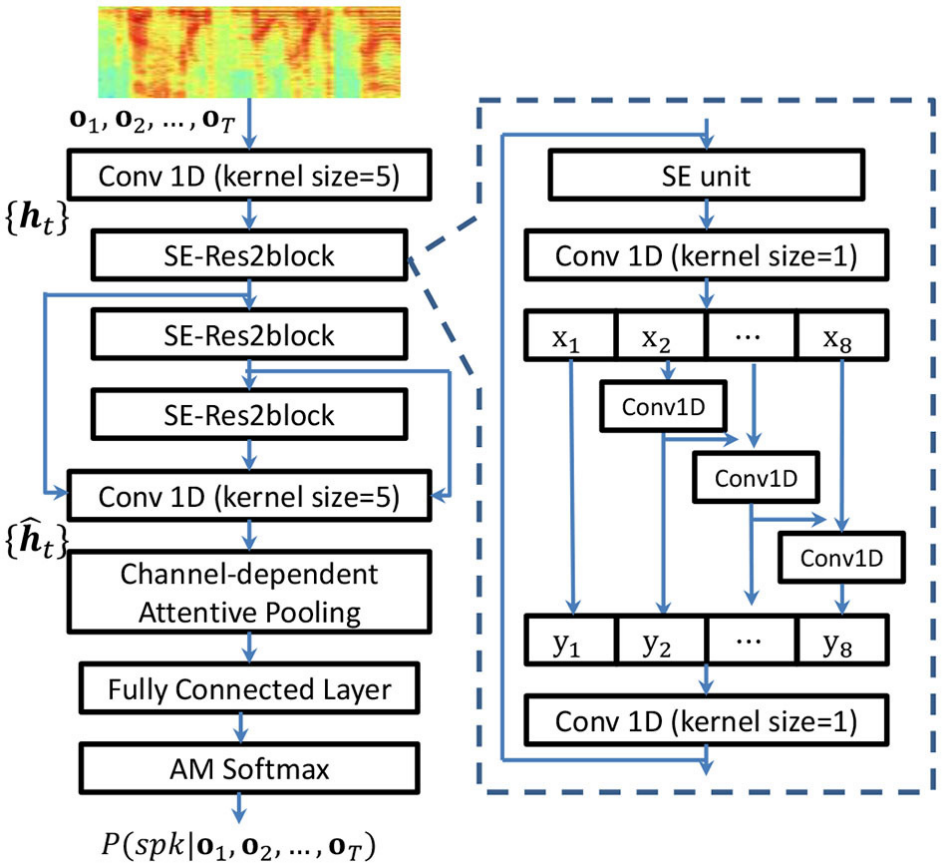
Structure of the CE-Res2Net and the SE-based Res2block. ${\left\{h_{t}\right\}}_{t=1}^{T}$ and ${\left\{{\hat{h}}_{t}\right\}}_{t=1}^{T}$ denote the frame-level features at different levels. *T* is the utterance length. ${\hat{h}}_{t}$ denotes the last frame-level convolutional layer's output.

The channel-interdependence enhanced Res2Net (CE-Res2Net; Zhao and Mak, 2021) was designed for tackling the problems of environmental noise and reverberation distortion in far-field speaker verification. Because the same problems exist in MoCA recordings, in this work, we applied CE-Res2Net for speaker embedding. The configuration of the CE-Res2Net is shown in Figure 3. The squeeze-and-excitation (SE) unit (Hu et al., 2018) is placed before the convolutional operations of the Res2block, which rescales the channel activations and facilitates the convolutional operations to learn multi-scale features.

In conventional speaker embedding, a self-attentive pooling layer (Okabe et al., 2018; Zhu et al., 2018) assigns a weight *e*_*t*_ for each frame-level vector ${\hat{h}}_{t}\in {\mathbb{R}}^{C}$, where *C* is the number of channels in the last convolutional layer. The weights *e*_*t*_'s are the output of a trainable network whose input is ${\hat{h}}_{t}$'s. However, this kind of mechanism assumes that all channels are of equal importance. To explore the importance of individual channels, the CE-Res2Net uses channel-dependent attentive pooling (Desplanques et al., 2020) to compute a scalar score *e*_*t, c*_ for each channel and each frame-level vector ${\hat{h}}_{t}$ at the last convolutional layer's output shown in Figure 3. Therefore, given ${\hat{h}}_{t}$, the attention network computes *e*_*t, c*_ by Equation (6):


(6) $$e_{t,c}=\;\upsilon_{c}^{T}f(W{\hat{h}}_{t}),\;c=1,\ldots ,C,$$

where **υ**_*c*_ and ***W*** are trainable parameters and *f*() is a non-linear function such as ReLU. *e*_*t, c*_ is then normalized across time by a softmax function in Equation (7):


(7) $$\alpha_{t,c}=\frac{\exp(e_{t,c})}{\sum_{\tau =1}^{T}\exp(e_{\tau ,c})},\;c=1,\ldots ,C.$$

Given a set of channel-dependent weights α_*t, c*_, the weighted average of channel *c* can be obtained using Equation (8):


(8) $${\hat{\mu }}_{c}=\sum_{t=1}^{T}\alpha_{t,c}{\hat{h}}_{t,c}.$$

The weighted mean vector is $\hat{\mu }={\left[{\hat{\mu }}_{1},{\hat{\mu }}_{2},\ldots ,{\hat{\mu }}_{C}\right]}^{T}$. Similar to self-attentive pooling, the elements of the weighted standard deviation vector $\hat{\sigma }={\left[{\hat{\sigma }}_{1},{\hat{\sigma }}_{2},\ldots ,{\hat{\sigma }}_{C}\right]}^{T}$ can be computed by Equation (9):


(9) $${\hat{\sigma }}_{c}=\sqrt{\frac{1}{T}\sum_{t=1}^{T}\alpha_{t,c}{\hat{h}}_{t,c}{\;}^{2}-{\hat{\mu }}_{c}{\;}^{2}},\;c=1,\ldots ,C.$$

By concatenating the weighted mean vector $\hat{\mu }$ and the weighted standard deviation vector $\hat{\sigma }$, the output of the channel-dependent attention pooling is obtained.

#### 2.2.2 LSTM-based scoring model

Although PLDA scoring is a prominent method for quantifying the similarity between short speech segments (typically 1.5 s) in speaker diarization systems, each PLDA score is based on the speaker embeddings of two short segments only, ignoring the remaining segments in a conversation. Because of the nature of MoCA sessions, each speaker will likely produce a consecutive sequence of short segments in an assessment session, i.e., neighboring segments have a higher chance of being produced by the same speaker. Therefore, instead of treating the segments independently, as in PLDA scoring, we should have a more holistic view of the segments. This notion leads to the LSTM-based scoring (Lin et al., 2019), which aims to capture the sequential information across the segments.

Given a conversation, we obtain a sequence of speaker embeddings $X=\{x_{1},\ldots ,x_{t},\ldots ,x_{T}\}$, where **x**_*t*_ represents the *t*-th segment's embedding and *T* is the number of segments. Each embedding, say **x**_*t*_, is concatenated with all the other embeddings to form a vector sequence of twice the dimension, as in Equation (10):


(10) $${ℋ}_{t}=\left\{\begin{array}{c} \left[\begin{array}{c} x_{t}\\ x_{1} \end{array}\right]\begin{array}{c} ,\ldots , \end{array}\left[\begin{array}{c} x_{t}\\ x_{t} \end{array}\right]\begin{array}{c} ,\ldots , \end{array}\left[\begin{array}{c} x_{t}\\ x_{T} \end{array}\right] \end{array}\right\}.$$

To exploit the temporal information in $X_{t}$, we feed it to a bidirectional-LSTM network (Huang et al., 2015) to produce the output, as in Equation (11):


(11) $$\begin{array}{l} S_{t}=f_{\text{LSTM}}\left(\begin{array}{c} \left[\begin{array}{c} x_{t}\\ x_{1} \end{array}\right]\begin{array}{c} ,\ldots , \end{array}\left[\begin{array}{c} x_{t}\\ x_{t} \end{array}\right]\begin{array}{c} ,\ldots , \end{array}\left[\begin{array}{c} x_{t}\\ x_{T} \end{array}\right] \end{array}\right)\\ \;=[S_{t1},\ldots ,S_{tt},\ldots ,S_{tT}]. \end{array}$$

**Figure 4:**
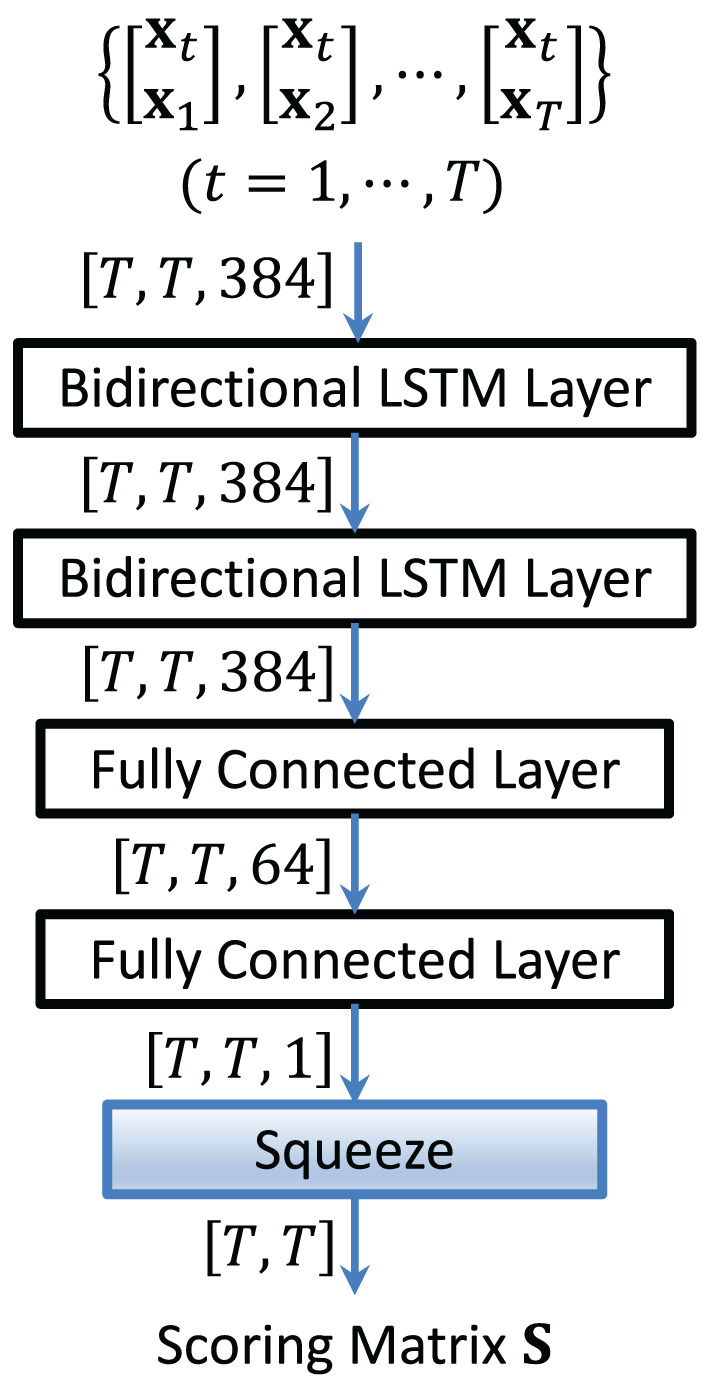
Structure of the LSTM-based scoring model. It aims to capture the sequential information in the concatenated vectors.

The vectors **S**_*t*_, *t* = 1, …, *T*, are then stacked row-wise to form a scoring matrix **S**. Figure 4 shows the structure of an LSTM-based scoring model. By using $H_{t}$ in Equation (10) as the input to the LSTM, each LSTM score in **S**_*t*_ depends not only on two embeddings but also on all other embeddings in the conversation and the sequential information in the concatenated vectors.

The basic idea of the method is to learn a reference similarity matrix, comprising blocks of ones and zeros. A “1” in the (*i, j*) entry of the reference matrix means that the *i*-th and *j*-th segments are produced by the same speaker; otherwise, it is a “0.” The matrix is formed from the speaker labels and timestamps of who spoke when in the training data, which is used as the labels for training the LSTM network.

In Lin et al. (2019), a *K*-fold cross-validation was applied to the Callhome dataset because timestamped speaker labels are available in Callhome. LSTM scoring can leverage the timestamp information about who spoke when in the training data. The diarization performance and the impact of utilizing both speaker labels and timestamp information are revealed in Section 3.

#### 2.2.3 Comprehensive scoring model

**Figure 5:**
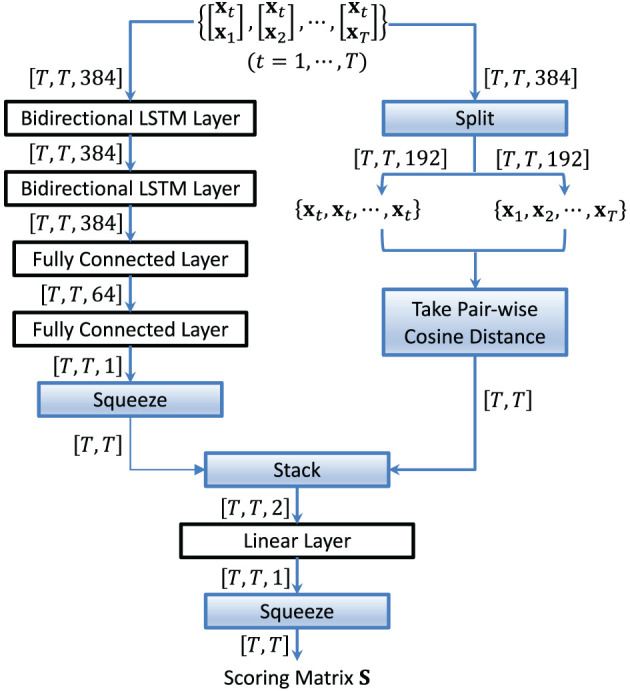
Structure of the comprehensive scoring model. It extends the sequential comparisons in the LSTM-based scoring model by incorporating cosine similarity into the score computation.

The cosine similarity and PLDA focus on pairwise comparisons, while the LSTM-based scoring captures the sequential structure in audio signals. To further enhance the similarity measure, in this work, we propose forming a similarity matrix that contains both sequential and pairwise information across the embeddings. The structure of a comprehensive scoring model is shown in Figure 5. The left branch computes the similarity scores between one embedding **x**_*t*_ and the whole embedding sequence X, and the right branch calculates the cosine similarity between any two embeddings in the sequence X. The scoring matrix is then obtained according to a weighted sum of the LSTM-based scores and cosine similarity scores. The pairwise and sequential scorings combination can be implemented using Equation (12):


(12) $$\begin{array}{l} S_{t}=R_{\text{L}}⊙f_{\text{LSTM}}\left(\begin{array}{c} \left[\begin{array}{c} x_{t}\\ x_{1} \end{array}\right]\begin{array}{c} , \end{array}\left[\begin{array}{c} x_{t}\\ x_{2} \end{array}\right]\begin{array}{c} ,\ldots , \end{array}\left[\begin{array}{c} x_{t}\\ x_{T} \end{array}\right] \end{array}\right)\\ \;+R_{\text{C}}⊙\text{cosine}\left(\begin{array}{c} \left\{x_{t},x_{t},\ldots ,x_{t}\},\{x_{1},x_{2},\ldots ,x_{T}\right\} \end{array}\right), \end{array}$$

where **R**_L_ and **R**_C_ contain the *T*-dimensional weights of the two scoring methods and ⊙ is the elementwise product.

#### 2.2.4 Spectral clustering

Spectral clustering (SC) can be viewed as graph cuts (Luxburg, 2007). The basic idea is to use the spectrum (eigenvalues) of an affinity matrix to perform dimension reduction. The general process of spectral clustering consists of three steps. First, a similarity graph based on all data points is constructed. Second, the data points are embedded on a low-dimensional space (spectral embedding), using the eigenvectors of the graph Laplacian. Third, a classical clustering algorithm (e.g., *K*-means) is applied to partition the embeddings.

Specifically, given a scoring matrix **S**∈ℝ^*n*×*n*^ with elements *S*_*ij*_≥0 and *S*_*ii*_ = 0 ∀*i*, we consider *S*_*ij*_ as the weight of the edge between nodes *i* and *j* in an undirected graph. Then, we compute a Laplacian matrix **L** = **D**−**S** and perform the following normalization using Equation (13):


(13) $$L_{\text{norm}}=D^{-\frac{1}{2}}LD^{-\frac{1}{2}},$$

where **D** is a diagonal matrix with $D_{ii}=\sum_{j}S_{ij}$. Next, we select the number of clusters *K* and take the *K* smallest eigenvalues λ_1_, …, λ_*K*_ and their corresponding eigenvectors **u**_1_, …, **u**_*K*_ from **L**_norm_ to form a matrix $U=[u_{1},\ldots ,u_{K}]\in {\mathbb{R}}^{T\times K}$ using **u**_1_, …, **u**_*K*_ as columns. Finally, we apply the *K*-means algorithm to cluster row vectors **y**_1_, …, **y**_*T*_ in **U** to form *K* classes, where **y**_*i*_∈ class *j* indicates that segment *i* belongs to speaker *j*.

Spectral clustering requires a similarity matrix comprising similarity scores among all speech segments in a conversation. In this work, SC is based on the cosine similarity scores and LSTM scores.

### 2.3 Experimental setup

#### 2.3.1 MoCA cantonese speech corpus

**Figure 6:**
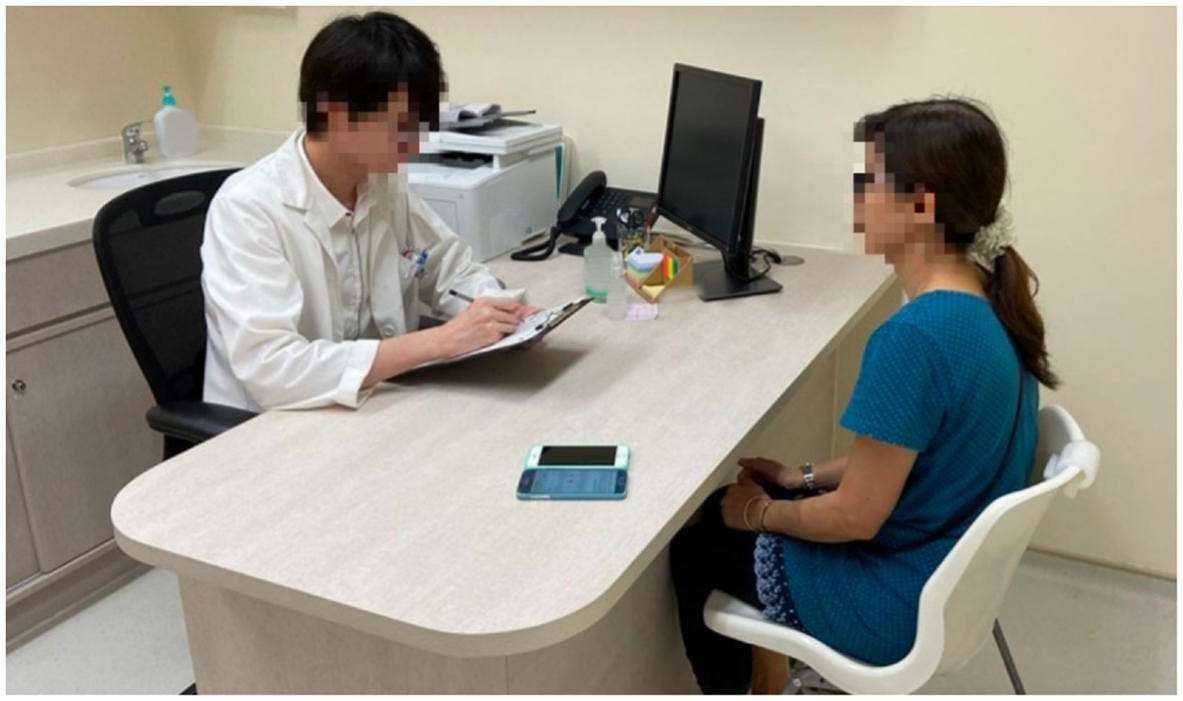
Collection of the JCCOCC–MoCA cantonese speech corpus.

The JCCOCC Montreal Cognitive Assessment (MoCA) Cantonese Speech corpus was collected by the CUHK Jockey Club Centre for Osteoporosis Care and Control. In the corpus, a MoCA test was conducted for each participant. There are 469 participants (both genders), each having an interactive spoken dialog session with an assessor. The average duration of the sessions is 26 min. The participants cover an age range of 72–100. The recordings were captured in a quiet office by two smartphones (iPhone 6 and Samsung Galaxy S6) placing at a distance from the participant, as shown in Figure 6. All of the 469 conversations were used in this work.

#### 2.3.2 Evaluation data

Among the 469 MoCA recordings, 256 (named MoCA-256) have been manually transcribed, and they were used for evaluating the performance of different diarization systems. The total duration of the evaluation data is 103.5 h, of which the speech duration of the assessors and the participants are 33.8 and 18.6 h, respectively.

#### 2.3.3 Training data for speaker embedding networks

In the experiments, the x-vector extractors and the CE-Res2Net were trained on the National Institute of Standards and Technology (NIST) Speaker Recognition Evaluations (SREs) and the Switchboard (SWB) datasets, including SRE 2004, 2005, 2006, 2008, SWB2 Phases 1, 2, and 3, SWB Cellular1, and SWB Cellular2. To obtain robust embeddings for diarization, we followed the data augmentation procedure in the Kaldi recipe and roughly doubled the size of the original clean data, i.e., using the room impulse responses (RIR; Ko et al., 2017) and the MUSAN datasets (Snyder et al., 2015) to create room reverberation and additive noise, respectively. Note that short utterances (number of frames < 400) and speakers with < 8 utterances were excluded. The statistics of the data for training the speaker embedding networks are shown in Table 1. We followed the Kaldi's Callhome recipe^1^ to train the speaker embedding networks.

**Table 1:** Source of data for training the x-vector extractor and the CE-Res2Net speaker-embedding network.

**Data source**	**No. of speakers**	**No. of hours**	**No. of utterances**
SRE 2004–2008 SWB and augmentation	4,979	2,789	62,151 (clean) 184,533 (augmentation)

#### 2.3.4 Training data for scoring models

To investigate the performance of different similarity measures, in addition to the 256 transcribed recordings, we also utilized the remaining unlabeled^2^ 213 MoCA recordings (called MoCA-213) as in-domain data to train the scoring models. Because information of speaker-turn timestamps is required for training the LSTM-based scoring models, we hypothesized the timestamped speaker labels of MoCA-213 in our experiments. In addition, we also used the Callhome portion of NIST SRE 2000 as out-of-domain data for training. Callhome,^3^ comprising 500 sessions with a total duration of 18 h, is a significant resource of telephone speech. The number of speakers per session varies from 2 to 7. Note that timestamped speaker labels are available in Callhome. Therefore, we used it to train both the LSTM-based and PLDA scoring models for performance comparison.

To train the LSTM models, we partitioned a long conversation into 300-s non-overlapping blocks and created a *T*×*T* reference matrix for each block. In our experiments, *T* in Equation (11) was set to 400. Because each speaker-embedding vector represents 0.75 s, the duration of each block in the long utterances is 400 × 0.75 = 300 s. The partitioning is to ensure enough temporal information in the blocks without excessive burden on computation resources. During scoring, the same partitioning was applied to the test conversations.

#### 2.3.5 Experimental settings

For SRE and SWB data, we used Kaldi's energy-based voice activity detection (VAD) to remove silence regions. For the JCCOCC–MoCA data, we used the ASpIRE speech activity detector (SAD).^4^ The reason for using two different VADs is that SRE and SWB contain clean telephone conversations. The signal-to-noise ratios are very high, and Kaldi VAD can do a good job. On the other hand, the interactive dialogs in JCCOCC–MoCA were collected by smartphones placing far away from the participants, causing lower signal-to-noise ratio. As a result, a DNN-based VAD that is more robust to noise was used for silence removal.

A sliding window of 1.5 s with 0.75 s shift was used to extract the embeddings in the speech regions of each conversation. Speech regions < 0.5 s were ignored. For each segment (or embedding), we computed a sequence of 23-dimensional MFCCs using a sliding window of 25 ms with a frameshift of 10ms; the MFCCs were then presented to the speaker embedding network to extract a speaker embedding vector.

We followed the configuration of CE-Res2Net described in Zhao and Mak (2021). One hundred and ninety-two-dimensional speaker embeddings were extracted from the affine layer's output after the statistics pooling layer. In addition, the LSTM-based scoring model in our experiments consists of two Bi-LSTM layers (384–384), followed by two dense layers (64–1). Each Bi-LSTM layer has 384 nodes including 192 forward nodes and 192 backward nodes. The first dense layer has 64 nodes with ReLU non-linearity. The output layer has one node with sigmoid non-linearity, which gives similarity scores between 0 and 1.

In general, a stopping threshold is needed in the clustering algorithms. However, because the number of speakers per recording is known, such stopping threshold is not needed in our case.

#### 2.3.6 Performance metrics

We reported the diarization error rate (DER; Fiscus et al., 2006) of different systems, which is a common performance metric based on comparing the .rttm file (hypothesis) generated by a diarization system with the ground-truth .rttm file (annotated). DER is the sum of the duration of missed speech (MS), false alarm (FA), and speaker error (SE) divided by the total duration, as computed in Equation (14):


(14) $$\begin{array}{l} \text{DER}=\frac{\text{Dur(MS) + Dur(FA) + Dur(SE)}}{\text{Total Duration of Reference Speech}}. \end{array}$$

In accordance with other studies (Anguera et al., 2012; Garcia-Romero et al., 2017; Lin et al., 2019), we allowed a non-scoring collar of 0.25 s around the reference segment boundaries and ignored the overlapped segments. Because MS and FA are caused by VAD errors, we may use SE to compare performance if the same VAD was used for all systems.

## 3 Results

This section first demonstrates the effectiveness of multi-scale channel inter-dependence speaker embedding as front-end speaker representation. Next, we conduct three baseline systems based on the x-vector–PLDA–AHC framework. Finally, we investigate the diarization performance achieved by different back-end scoring approaches.

### 3.1 Effect of multi-scale feature aggregation

**Figure 7:**
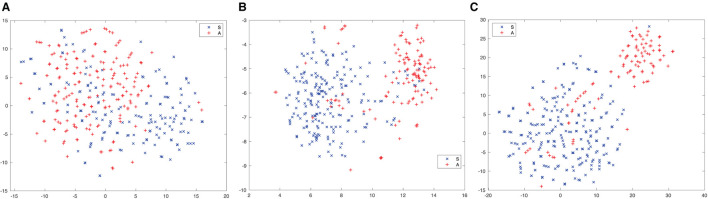
t-SNE plots of speaker embeddings extracted from different speaker embedding networks. The red and blue markers refer to the speaker embeddings of an assessor (A) and a subject (S), respectively. It is clear that in **(C)**, the two classes are well-separated, and the number of misclassifications is smaller. **(A)** Kaldi x-vectors. **(B)** Res2Net. **(C)** CE-Res2Net.

To show the effect of inter-dependence between channels and channel-dependent attention, we presented ten minutes of a MoCA recording, including utterances of an assessor (A) and a subject (S), to different embedding extractors, i.e., Kaldi x-vectors, Res2net, and CE-Res2Net. The t-SNE (Maaten and Hinton, 2008) was applied to the speaker emdeddings. The results are shown in Figure 7 for Classes A and S. We can see that there is no obvious clusters in the Kaldi x-vectors, and the clusters become more distinct in Res2Net and CE-Res2Net. Moreover, fewer embeddings are misclassified in Figure 7C. This means that the CE-Res2net achieves better performance by introducing SE units and channel-dependent attention into the network.

### 3.2 Advanced baseline systems

We constructed three baseline systems based on the evaluation set (MoCA-256), i.e., using a Kaldi x-vector network, a Res2Net and a CE-Res2Net for embedding extraction, PLDA for similarity measures, and AHC for clustering. The SRE data (without augmentation) was used to train the PLDA models. Table 2 shows the diarization performance of the baseline systems. The results based on MoCA-256 show that CE-Res2Net can produce the best embeddings and achieve the lowest DER among the three embedding extractors. Therefore, the t-SNE plots in Figure 7 are supported and we only used CE-Res2Net for embedding extraction in subsequent experiments.

**Table 2:** Diarization performance of the baseline systems (based on MoCA-256).

**System architecture**	**Performance metrics (%)**			
	**DER**	**MS**	**FA**	**SE**
Kaldi x-vector + PLDA + AHC	7.37	2.7	1.8	2.9
Res2Net + PLDA + AHC	7.03	2.7	1.8	2.6
CE-Res2Net + PLDA + AHC	6.86	2.7	1.8	2.4

The SRE data was used to train the PLDA models.

### 3.3 Diarization performance comparison

To evaluate the diarization performance of different similarity measures, we replaced the conventional PLDA scoring with the LSTM-based scoring and the proposed comprehensive scoring. We employed in-domain (e.g., MoCA) and out-of-domain (e.g., Callhome) data for training the scoring models. Moreover, the in-domain data with hypothesized labels were utilized. We also applied different clustering algorithms (e.g., AHC and SC) for comparisons. The diarization performance achieved by different similarity measures (e.g., PLDA, LSTM, and LSTM+Cosine) based on different training data and label information is given in Table 3. Cosine+AHC is used as a baseline for comparison; it achieved a speaker error rate of 3.7%.

**Table 3:** Diarization performance achieved by different similarity measures based on different training data and label information.

**Similarity measure**					
**Case**	**Model**	**Training data**	**Label Info**.	**Clustering**	**SE (%)**
1	PLDA	Callhome	Speaker labels (Ground truth)	AHC	3.3
	LSTM	Callhome	Timestamped speaker labels (Ground truth)	AHC	2.4
2	LSTM	Callhome	Timestamped speaker labels (Ground truth)	SC	2.1
	LSTM+Cosine	Callhome	Timestamped speaker labels (Ground truth)	AHC	2.0
3	LSTM+Cosine	Callhome	Timestamped speaker labels (Ground truth)	SC	1.7
	LSTM	MoCA-256	Timestamped speaker labels (Ground truth)	AHC	1.5
4	LSTM	MoCA-256	Timestamped speaker labels (Ground truth)	SC	1.3
	LSTM+Cosine	MoCA-256	Timestamped speaker labels (Ground truth)	AHC	1.5
5	LSTM+Cosine	MoCA-256	Timestamped speaker labels (Ground truth)	SC	**1.2**
	LSTM	MoCA-213	Timestamped speaker labels (Hypothesized)	AHC	1.8
6	LSTM	MoCA-213	Timestamped speaker labels (Hypothesized)	SC	1.7
	LSTM+Cosine	MoCA-213	Timestamped speaker labels (Hypothesized)	AHC	1.6
7	LSTM+Cosine	MoCA-213	Timestamped speaker labels (Hypothesized)	SC	1.5

Note that since the same voice activity detection method was applied across all cases, the missed speech and false alarm error rates are 2.7 and 1.8%, respectively. Bold values is the best performance in each column.

In Case 4 and Case 5, the LSTM and LSTM+Cosine models were trained using the labeled in-domain data. Specifically, 5-fold cross-validation was conducted to estimate the performance, i.e., the evaluation set (MoCA-256) was randomly partitioned into five equal-sized subsets. A subset was retained as the test data while the remaining four subsets were used for training the LSTM and LSTM+Cosine models. The procedure was repeated five times, and each subset was used once as the test data. After that, the five-fold test results were combined to calculate the DER. In contrast, the labeled out-of-domain (Callhome) and unlabeled in-domain (MoCA-213) data were used to train the LSTM and LSTM+Cosine models, respectively, as shown in Cases 1–2 and Cases 6–7. Note that the corresponding labels (i.e., timestamped speaker labels) were hypothesized by the advanced baseline system that uses CE-Res2Net as the speaker embedding network and PLDA for scoring (see Section 3.2). Specifically, a speaker error rate of 2.3% on the unlabeled in-domain MoCA-213 dataset was achieved. Therefore, the training manner in Case 6 and Case 7 is semi-supervised.

The results based on the evaluation set (MoCA-256) demonstrate that, in Cases 2–7, spectral clustering outperforms AHC; moreover, comparing the LSTM and LSTM+Cosine models, the latter achieves better performance. Specifically, with ground-truth timestamped speaker labels, the in-domain five-fold cross-validations in Case 4 and Case 5 produce lower DERs than the other cases. The lowest DER (5.68%) is obtained by using LSTM-Cosine scoring and spectral clustering in Case 5. In Case 1, the lack of training data in Callhome may cause poorer performance than the baseline in Table 2.

Case 2 and Case 3 achieve performance comparable with the baseline even with less training data, which demonstrates the benefit of using the timestamp information in Callhome. We utilized unlabeled in-domain data to train the LSTM and LSTM+Cosine models in Case 6 and Case 7, respectively, and the DER of both cases are lower than that of the baseline in Table 2, demonstrating the effectiveness of learning representations from in-domain data. Note that, the unlabeled in-domain data (MoCA-213) cannot be used to train the PLDA model because we cannot be sure the same speaker exists in other MoCA recordings.

## 4 Discussion

### 4.1 Compared with conformer-based diarization systems

Recently, both Transformer and Conformer have demonstrated outstanding performance in NLP and speech tasks. In particular, Liu et al. (2021) introduced an end-to-end diarization system based on the Conformer, a combination of convolutional networks and Transformer to model the short-term and long-term dependence in speech. The authors further enhance the diarization performance by adding a spectral-augmentation layer and sub-sampling layer before the Conformer blocks. Although the system outperforms those based on Transformer, it shows limitations in generalizing to real conversations. Jung et al. (2023) studied speaker embedding extractors for diarization purpose. They developed special evaluation protocols and data augmentation methods to improve Conformer-based diarization in complex scenarios. However, Conformer and Transformer models require large training sets to achieve good performance, and their training becomes prohibitory expensively for long utterances. Given the small size of the MoCA dataset, the proposed approach, which uses a speaker-embedding network to model the local dependence within a short speech segment and an LSTM-based model to capture the long-term dependence in the short speech segments, offers a more practical solution.

### 4.2 Diarization and dementia classification

Diarization and dementia classification are two distinct tasks within the realm of speech and audio processing. Diarization refers to the process of separating speakers in an audio recording, while dementia classification aims to identify whether a speaker has dementia based on their speech patterns. Although the two tasks are separate, they can be related in the context of analyzing speech data from individuals with and without dementia. The diarization process serves as a preprocessing step, separating and labeling the speech segments for each speaker. Once the audio data have been segmented, the dementia classification model can be applied to the participants' data to determine whether they exhibit signs of dementia.

**Figure 8:**
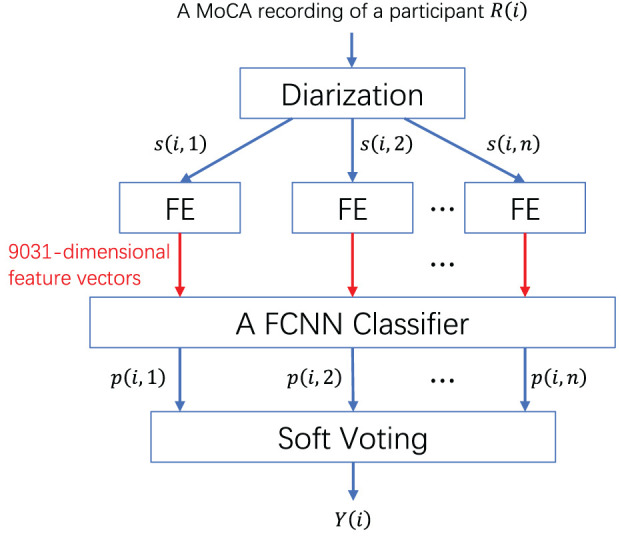
System architecture for detecting dementia from spontaneous Cantonese speech.

In this section, we explore the possibility that diarization outcomes, achieved through various similarity measures, may affect the performance of dementia classification. The procedure is illustrated in Figure 8. For a given MoCA recording of a participant *R*(*i*), we segmented the recording based on the diarization results (i.e., timestamp information of different speakers) and extracted *n* speech segments belonging to the participant. Feature extraction (FE; Ke et al., 2022) was then conducted at the segment level. Specifically, four paralinguistic feature sets, ComparE, Emobase, IS10, and extended Geneva Minimalistic Acoustic Parameter Set (eGeMAPS) were extracted, respectively. The ComParE feature set (Eyben et al., 2010) consists of some generic low-level descriptors (LLDs) and their statistical functionals to characterize emotion, including energy, spectral characteristics, mel-frequency cepstral coefficients (MFCCs), and voicing-related LLDs. The Emobase feature set (Eyben et al., 2010) is specifically designed for emotion recognition, comprising MFCCs, fundamental frequency (F0), F0 envelope, line spectral pairs (LSP), etc. The IS10 (Schuller et al., 2010) is a feature set utilized for emotion recognition and bipolar disorder recognition, including PCM loudness, log Mel-frequency bands, line spectral frequency pairs, F0 envelope, voicing probability, jitter, and shimmer. The eGeMAPS (Eyben et al., 2016) contains 88 features that are selected based on their potential for characterizing physiological changes in voice production. Regarding dementia detection, Haider et al. (2020) conducted a comparative study using ComParE, Emobase, and eGeMAPS. Additionally, these feature sets were benchmarked for AD recognition performance in the AD Recognition Through Spontaneous Speech Challenge (ADReSS; Luz et al., 2020), where the IS10 feature set served as the baseline for AD recognition in the AD2021 competition (Qin et al., 2021). These four feature sets were concatenated into a high-dimensional feature vector with a total dimension of 9,031. Next, we trained a fully-connected neural network (FCNN) with the structure 9031–2048–128–2 to perform classification at the segment level, producing a prediction score *p*(*i, n*) for each segment. A dropout layer with a dropout rate of 0.5 was added after each hidden layer in the network to mitigate the risk of overfitting. The activation function for the hidden layers is ReLU, while the activation function for the last layer is softmax. An Adam optimizer with a learning rate of 0.001 was used to optimize the network' parameters. The batch size was set to 4, and the number of epochs was set to 10. Finally, soft voting was employed to average the prediction scores, yielding the label *Y*(*i*) (e.g., normal or possible dementia) for the recording *R*(*i*).

In practice, for dementia detection, we grouped mild neurocognitive disorders (NCDs), mild cognitive impairment (MCI), and major NCDs into a single category, termed possible dementia. We excluded certain participants who lacked classification labels in the corpus. As a result, the MoCA-213 dataset comprises 164 healthy participants and 36 patients with possible dementia, while the MoCA-256 dataset includes 201 healthy participants and 43 patients with possible dementia. Table 4 presents the combination of possible dementia type and shows the number of participants in the two classes. We utilized MoCA-213 with hypothesized timestamped speaker labels as training data for the FCNN classifier. We employed MoCA-256 as the test data to evaluate different similarity measures. Additionally, we incorporated MoCA-256 with ground truth timestamped speaker labels for performance comparison. Due to the imbalanced distribution of classes, the overall performance of different cases was compared using not only classification accuracy but also *F*1 scores. The *F*1 scores serve as an aggregated measure of performance by computing the harmonic mean of precision and recall. The dementia classification performance achieved by different similarity measures is presented in Table 5.

**Table 4:** Combination of the category termed “possible dementia” and the number of participants in the two classes.

**Dataset**	**No. of normal**	**No. of possible dementia**		
		**MCI**	**NCDs**	**Major NCDs**
MoCA-213	164	36		
MoCA-256	201	43		

**Table 5:** Dementia detection performance achieved by different similarity measures based on different scoring models and ground truth timestamp information.

	**Diarization**		**Dementia detection**			
**Case**	**Evaluationset**	**Similarity (scoring model)**	**Speaker error (%)**	**Accuracy (%)**	**Precision (%)**	**Recall (%)**	**F1(%)**
1	MoCA-213	LSTM+Cosine	1.5	78.0	56.7	54.1	54.3
2	MoCA-256	LSTM+Cosine	1.2	**77.9**	56.4	54.2	54.5
3	MoCA-256	LSTM	1.3	77.5	55.4	53.6	53.8
4	MoCA-256	Timestamp information (Ground truth)	0	76.5	**57.4**	**56.5**	**56.8**

MoCA-213 with hypothesized timestamped speaker labels were utilized to train the classifiers for all cases. In Case 1, five-fold cross-validation was conducted to evaluate the performance. Bold values is the best performance in each column.

Table 5 reveals that the highest F1 score (56.8%) is achieved in Case 4, suggesting that the dementia classifier achieves optimal performance when utilizing ground truth timestamp information for speaker diarization. In Cases 2 and 3, the classification performance of MoCA-256 with LSTM+Cosine surpasses that of MoCA-256 with LSTM, indicating that superior diarization results, i.e., lower DER, positively impact dementia classification outcomes. This means that the proposed comprehensive similarity measure (LSTM+Cosine) benefits dementia classification. Moreover, the classification performance in Cases 2 and 3 is comparable to that of Case 4, implying that our predicted timestamp information can also be utilized for dementia detection, even though it results in a slight decrease in performance compared to using ground truth timestamp information.

## 5 Conclusions

In this paper, we propose a speaker diarization system for speech-based MoCA recordings. The system incorporates a CE-Res2Net embedding extractor and a comprehensive scoring model. To obtain better speaker embeddings, the CE-Res2Net exploits the inter-dependence between the channels in the last convolutional layer. The comprehensive scoring model, which learns a weighted sum of the LSTM-based and cosine similarities, substitutes the conventional PLDA for similarity measures.

Experimental results based on MoCA data show that by leveraging both speaker labels and timestamps, the LSTM scoring model trained on in-domain or out-of-domain data performs better than the PLDA model. Moreover, by incorporating pairwise cosine similarity, the proposed LSTM+Cosine scoring model can improve the diarization performance further. While LSTM+Cosine scoring requires the timestamp information about who spoke when in the training data, results show that the LSTM+Cosine model can tolerate some errors in the timestamps, suggesting that this scoring approach can leverage unlabeled training data via hypothesizing the timestamp information.

Speaker diarization is conducted by the first module to facilitate conversation-based screening of neurocognitive disorders (e.g., AD and MCI). Given that the raw MoCA recordings typically comprise interactive dialogues between the assessors and patients, the purpose of the diarization is to extract the patients' speech segments for further processing. Consequently, the proposed speaker-turn aware diarization, which can be applied to various feature extraction methods, serves as a tool for clinicians in the early detection of cognitive impairments. On the other hand, one limitation is its inability to perform online (real-time) diarization, due to the need for hypothesized timestamped labels as intermediate information. Moreover, the diarization accuracy could be impacted by varying recording conditions and background noise, which are commonly found in real-world clinical settings. Another limitation is that the method becomes computationally expensive when the conversation lasts for hours. However, this problem can be solved by manually partitioning the long recording into several short conversations.

## Data availability statement

The raw data supporting the conclusions of this article will be made available by the authors, without undue reservation.

## Ethics statement

The studies involving humans were approved by The Chinese University of Hong Kong. The studies were conducted in accordance with the local legislation and institutional requirements. Written informed consent for participation in this study was provided by the participants' legal guardians/next of kin. Written informed consent was obtained from the individual(s) for the publication of any potentially identifiable images or data included in this article.

## Author contributions

SX: Methodology, Software, Writing—original draft, Writing—review & editing, Conceptualization, Validation, Visualization. XK: Formal analysis, Writing—review & editing. M-WM: Supervision, Writing— review & editing, Conceptualization. KW: Resources, Writing—review & editing. HM: Funding acquisition, Supervision, Writing—review & editing. TK: Data curation, Writing—review & editing. JG: Funding acquisition, Writing—review & editing. JZ: Funding acquisition, Writing—review & editing. WT: Funding acquisition, Writing—review & editing. CC: Funding acquisition, Supervision, Writing—review & editing.

## Footnotes

^1^ https://github.com/kaldi-asr/kaldi/tree/master/egs/callhome_diarization/v2

^2^In this work, the term “unlabeled” means there is no annotation, such as speaker labels and timestamps of who spoke when in the dataset, i.e., it only has audio files.

^3^2000 NIST Speaker Recognition Evaluation (LDC2001S97), Disk-8.

^4^ https://kaldi-asr.org/models/m4
